# Physiological limits of capillary refill time: a human endotoxemia study

**DOI:** 10.1186/s13054-026-06247-8

**Published:** 2026-08-31

**Authors:** Hugo Gustavsson, Rani Toll, Sara Fahlander, Frida Meyer, Birgitta Ölwegård, Martin Hultman, Julie Lasselin, Joakim Henricson, Daniel Wilhelms

**Affiliations:** 1 Department of Biomedical and Clinical Sciences, Linköping University, Linköping, Sweden; 2 Clinical Department Emergency Medicine in Linköping, Region Östergötland, Linköping, Sweden; 3 Department of Biomedical Engineering, Linköping University, 581 83 Linköping, Sweden; 4 Department of Psychology, Stockholm University, Stockholm, Sweden; 5 Department of Clinical Neuroscience, Division of Psychology, Karolinska Institutet, Solna, Sweden; 6 Department of Clinical Neuroscience, Osher Center for Integrative Health, Karolinska Institutet, Stockholm, Sweden

## Abstract

**Background:**

The capillary refill test is a widely used bedside marker of tissue perfusion, but its reliability during acute inflammation is not well characterised. The guiding 3-s threshold rests on limited physiological validation and on visual assessment with high observer variability. How finger capillary refill time (CRT) behaves during acute inflammation remains unclear. Using quantitative capillary refill time (qCRT), which removes observer variability, we determined how finger qCRT behaves over an acute inflammatory episode in a controlled human endotoxemia model.

**Methods:**

In a double-blind, randomized, crossover study, 25 healthy volunteers received an intravenous bolus of *Escherichia coli* lipopolysaccharide (0.8 ng/kg) or saline placebo. Finger qCRT was measured by polarized reflectance imaging at baseline and 1.5, 3.5, and 5 h, and analysed with censored (Tobit) mixed-effects models adjusting for physiological covariates.

**Results:**

Finger qCRT followed a biphasic course that crossed the 3-s clinical threshold in both directions within a single inflammatory episode. From a baseline near 3.5 s, it was prolonged to 8.31 s at 1.5 h, with 14 of 25 reaching the 10-s ceiling, then shortened to 1.27 s at 5 h during persisting systemic inflammation. Estimated marginal contrasts against placebo were 4.13 s and − 3.10 s at these time points (both p < 0.0001). The early prolongation persisted after adjustment for physiological covariates, whereas the late shortening was attenuated after adjustment for concurrent thermoregulatory and haemodynamic covariates. Skin temperature was the strongest qCRT covariate, with each standard-deviation increase associated with a 1.62 s decreased qCRT. No formal mediation analysis was performed. Resting values were highly variable, with between-subject differences explaining a minority of total variance (intraclass correlation 0.30).

**Conclusions:**

Finger qCRT tracks the course of acute systemic inflammation but crosses the 3-s clinical threshold in both directions within a single episode. Because between-subject differences account for a minority of total variance and resting values depend strongly on skin temperature, a single value against a fixed threshold is unreliable, whereas change over time within a participant is informative. These findings in healthy participants are not directly generalizable to septic shock and require confirmation in prospective patient studies before informing clinical practice.

**Trial registration:**

ClinicalTrials.gov NCT06618716. Registered September 27, 2024.

**Supplementary Information:**

The online version contains supplementary material available at https://doi.org/10.1186/s13054-026-06247-8.

## Background

The capillary refill test is a widely used bedside assessment of tissue perfusion in emergency and critical care settings [1, 2]. Theoretically, it serves distinct roles: as a diagnostic marker and aid in triage [3–5], as a prognostic marker associated with mortality and organ dysfunction [1, 3, 6], and as a resuscitation target [1, 7–10]. Furthermore, it serves a role as a physiological variable, whose underlying mechanisms and dynamics remain an active area of research [11–14].

The test is limited by substantial inter- and intra-observer variability when assessed by the naked eye [15–17], and refill time is also known to vary with age, sex, and ambient temperature [16–20]. Consequently, standardized clinical protocols [9, 21] have been suggested to mitigate inter- and intra-observer variability, while quantitative capillary refill time (qCRT), measured using optical techniques such as polarized reflectance imaging, offers a more objective and reproducible alternative to visual estimation [17, 22, 23].

A 3-s CRT threshold is widely used in triage and for early warning scores [19, 24]. However, evidence supporting this threshold as a diagnostic cutoff for identifying shock, as was its original purpose when introduced by Beecher in 1947 [5], or to detect clinically significant peripheral hypoperfusion remains limited. In contrast, there is support for it as a resuscitation target and prognostic marker, where the latest literature is largely driven by the investigators of the ANDROMEDA-SHOCK 1 & 2 trials [6, 8–10, 12, 21, 25, 26].

However, septic shock involves complex overlapping pathophysiological mechanisms, and the isolated response of qCRT during systemic inflammation remains incompletely characterized. Controlled human endotoxemia provides a standardized experimental model to isolate the physiological kinetics of qCRT during acute, transient systemic inflammation in healthy individuals, unconfounded by vasopressor therapy, organ failure, or altered cardiac output [27–30]. While CRT probably reflects multiple determinants simultaneously, characterizing these responses helps delineate parts of the fundamental physiological determinants. Therefore, the primary objective of this study was to evaluate qCRT trajectories, using a Tobit mixed-effects modelling, following lipopolysaccharide (LPS) administration compared to placebo in an experimental double-blind, crossover study. As a secondary exploratory objective, we deployed a covariate adjusted model. In this second model we examined the relative contributions of physiological covariates (local skin temperature, body temperature, blood pressure, heart rate, and plasma interleukin-6 (IL-6)), to determine how thermoregulatory and inflammatory responses influence qCRT dynamics over time.

## Methods

### Study design and setting

This was a double-blind, within-subject, randomized, crossover, placebo-controlled study conducted at Linköping University Hospital, Sweden, between September 2024 and March 2025. Each participant attended two study visits, one with injection of intravenous lipopolysaccharide (LPS) and one with placebo, in randomized order. Visits were separated by at least two weeks for men and by approximately one month for women, to control for menstrual cycle–related hormonal effects. Ethical approval was obtained from the Swedish Ethical Review Authority (permit numbers 2023–07974-01 and 2024–06056-02). All participants provided written informed consent. The protocol was pre-registered (Clinicaltrials.gov: NCT06618716; registered September 27, 2024).

### Selection of participants

**Fig. 1:**
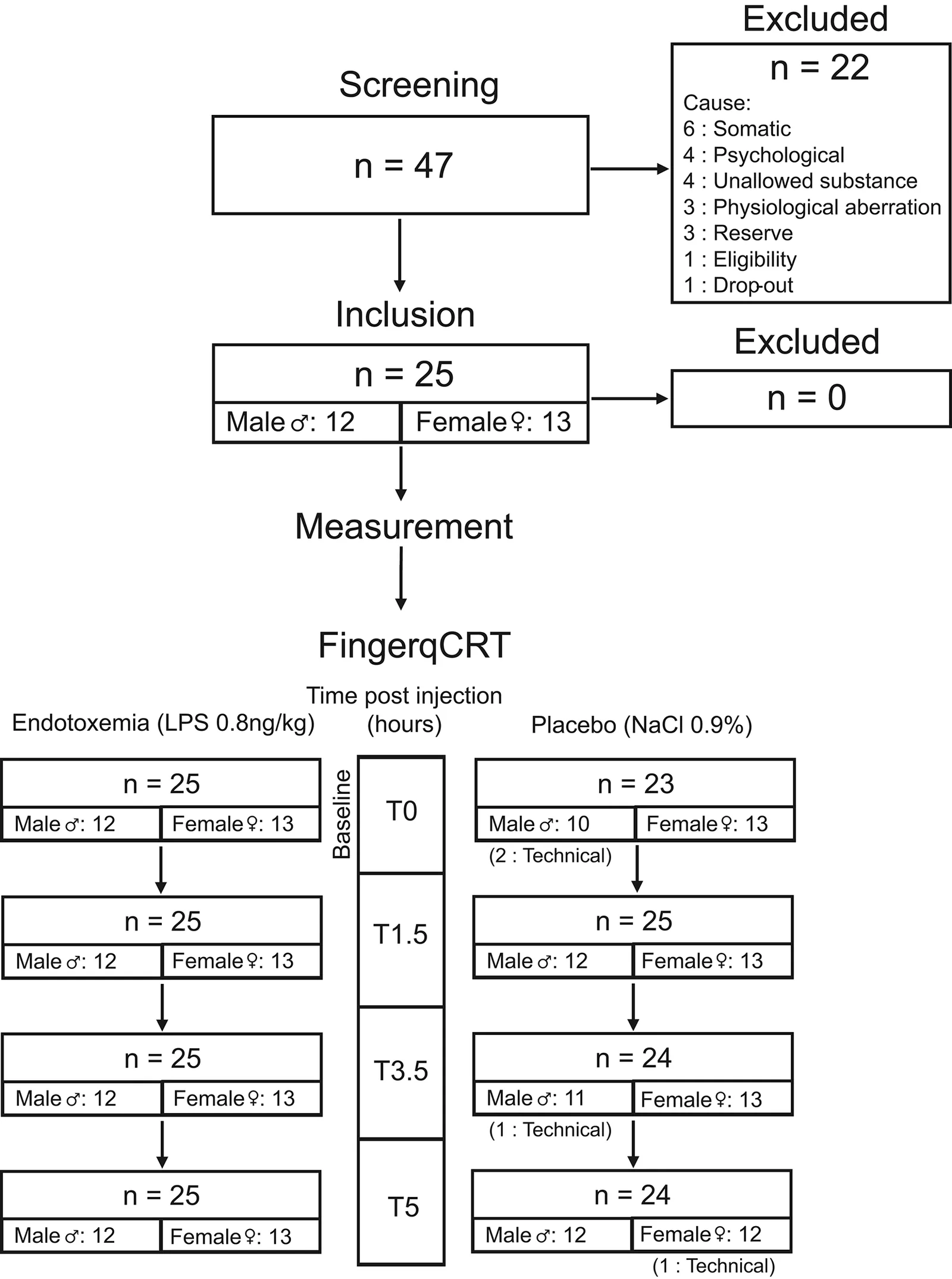
Flow diagram of the inclusion process, exclusions and data loss. Time points are given as T hours after injection and T0 corresponds to baseline measurements. Male and female distributions are presented for each step after inclusion and missing data is explained in parentheses (all data loss was assumed to be random and could be attributed to various technical issues)

We included healthy adults aged 18 to 40 years, body mass index (BMI) between 18.5 and 30 kg/m^2^, with Swedish social security number. Key exclusion criteria were a history of illness (somatic or psychiatric), current medication use (other than contraceptives), nicotine use, high-sensitivity C-reactive protein above 10 mg/L or other abnormal screening blood analysis, high blood pressure or other indicators of cardiovascular abnormalities, shiftwork within 14 days, or previous exposure to LPS in a study setting. A complete list of criteria is provided in Supplementary Materials. Of 47 screened volunteers, 25 were enrolled (13 females, 12 males, mean age: 23 years, *SD* = 4, mean BMI: 23.7 kg/m^2^, *SD* = 1.7). The main reason for exclusion was a pre-existing somatic or psychiatric condition discovered at screening (Fig. 1). All enrolled participants completed the study protocol (Fig. 1).

Sample size was estimated for a paired design assuming a minimum clinically relevant difference of 0.3 s (SD 0.3 s; Cohen's *d* = 1.0). At a two-sided α of 0.05, between 10 and 15 participants were required to achieve 80–95% power, respectively; to reach the upper bound of 95% power with a margin for dropout, a recruitment target of 17–18 participants were set. To further support secondary exploratory covariate analyses with adequate precision, and given that this was the first study doing CRT measurements during LPS challenge, we recruited 25 participants (in accordance with the ethical permit).

### Interventions

Participants received an intravenous bolus of *Escherichia coli* Lipopolysaccharide (LPS) (0.8 ng/kg; USP Endotoxin Reference Standard, Lot H0K354; imported to Sweden by Sigma-Aldrich Sweden AB) on one occasion and an equivolume placebo (0.9% NaCl) on the other occasion (randomized in a 1:1 ratio using a computer-generated randomization sequence). LPS was reconstituted from lyophilized powder to a final concentration of 32 ng/mL and independently verified for endotoxin content before the study (ALS Scandinavia AB, Danderyd, Sweden).

At this dose, LPS reliably induces a moderate systemic inflammatory response without excessive discomfort [27, 28, 31–33].

### Measurements

#### Primary outcome

Quantitative capillary refill time (qCRT) was measured at the right index finger pulp at four time points: baseline (pre-injection) and 1.5, 3.5, and 5 h post-injection. We used a standardized 10 N dynamometer for blanching pressure and a polarized reflectance imaging system (Tissue Viability Imaging [TiVi]; WheelsBridge AB, Linköping, Sweden) for quantification [22, 23]. The system estimates relative red blood cell concentration in the superficial dermal microvasculature (penetration depth ~ 500 µm) by analyzing backscattered polarized light in the red (600–700 nm) and green (500–600 nm) wavelength regions, sampled at 50 Hz with 10-ms output precision (reported as TiVi value) [23]. Baseline colour was recorded for 5 s, blanching pressure applied for 5 s, and refilling recorded for 10 s. The refill time was defined as the time for the compressed area to return to its baseline TiVi value, determined through post-processing. Times exceeding the 10-s recording window were assigned 10 s.

#### Covariates

Tympanic temperature was recorded every 30 min for 3 h, then hourly (Braun ThermoScan Pro 6000; Welch Allyn, USA). Skin temperature at the qCRT site was measured with an infrared pyrometer (Fluke 572; Fluke, Germany). Blood samples for plasma interleukin-6 (IL-6) were drawn at baseline, 0.5 h, and hourly thereafter for 6 h (Cobas e 801 immunoassay; Roche, USA; measuring range 1.5–5000 ng/L). Systolic and diastolic blood pressures were measured on the opposite upper arm using an automated brachial cuff (Omron M7; Omron Healthcare Co, Japan). Heart rate was measured by photoplethysmographic pulse oximetry on the left index finger (Nonin GO_2_; Nonin, USA). All measurements were performed in the same windowless room with controlled lighting (mean ambient temperature 21.8 °C, SD 0.6).

### Analysis

#### General

Models were fitted in R (version 4.5.1; R Core Team, 2025). To account for right-censoring at 10.0 s, censored mixed-effects (Tobit) models were implemented using the glmmTMB package. Statistical significance was defined as p < 0.05. Diagnostic plots (Residuals vs. Fitted and Q-Q plots) were inspected to ensure assumptions of normality and homoscedasticity were met. Figures were generated using GraphPad Prism (version 10.0.2) and ggplot2 in R.

#### Primary analysis (Model m0)

To test whether qCRT trajectories differed between endotoxemia and placebo over time, we used a Tobit mixed-effects model. This model included fixed effects for Condition (endotoxemia vs. placebo), Time point (baseline, T1.5, T3.5, T5), and their interaction, with subject-specific random intercepts. The model (m0) was defined as:

$$\rm{qCRT\hspace{0.17em}\sim \hspace{0.17em}Condition\hspace{0.17em}\times \hspace{0.17em}Timepoint\hspace{0.17em}+\hspace{0.17em}(1|Subject)}$$

To facilitate interpretation, the emmeans package was used to extract post-estimation Estimated Marginal Means (EMMs) and pairwise condition contrasts (Endotoxemia − Placebo) at each individual time point. The underlying fixed-effect parameter estimates from the model are provided in Supplementary Table 1. Intraclass correlation coefficients (ICC) were calculated from the random-intercept variance of this unadjusted model to quantify the proportion of variance attributable to stable between-subject differences.

#### Secondary exploratory analysis (Model m1)

To assess the robustness of the primary findings and the influence of physiological shifts, we fitted a covariate-adjusted Tobit mixed-effects model adding heart rate, systolic and diastolic blood pressure, tympanic temperature, local skin temperature, and plasma IL-6 concentrations. IL-6 was natural log-transformed (ln) prior to analysis to satisfy model assumptions.

All covariates were standardized using global z-scoring to improve model stability and allow for a direct comparison of effect sizes across different physiological scales. Consequently, the coefficients for these covariates (β) represent the estimated change in qCRT (in seconds) per one standard deviation increase in the covariate, with the intercept representing the qCRT of a participant with average physiological values (z = 0) at placebo baseline. The adjusted model (m1) was specified as:

$$\begin{gathered}\mathrm{q}\mathrm{C}\mathrm{R}\mathrm{T} \sim \mathrm{C}\mathrm{o}\mathrm{n}\mathrm{d}\mathrm{i}\mathrm{t}\mathrm{i}\mathrm{o}\mathrm{n} \times \mathrm{T}\mathrm{i}\mathrm{m}\mathrm{e}\mathrm{p}\mathrm{o}\mathrm{i}\mathrm{n}\mathrm{t} \\+\mathrm{z}\_\mathrm{T}\mathrm{y}\mathrm{m}\mathrm{p}\mathrm{T}\mathrm{e}\mathrm{m}\mathrm{p} + \mathrm{z}\_\mathrm{S}\mathrm{k}\mathrm{i}\mathrm{n}\mathrm{T}\mathrm{e}\mathrm{m}\mathrm{p}\\ + \mathrm{z}\_\mathrm{S}\mathrm{B}\mathrm{P} + \mathrm{z}\_\mathrm{D}\mathrm{B}\mathrm{P} + \mathrm{z}\_\mathrm{H}\mathrm{R} \\+ \mathrm{z}\_ln(\mathrm{I}\mathrm{L}6) + (1|\mathrm{S}\mathrm{u}\mathrm{b}\mathrm{j}\mathrm{e}\mathrm{c}\mathrm{t})\end{gathered}$$

Multicollinearity among covariates was assessed using Variance Inflation Factors (VIF). The full numerical parameter estimates for this adjusted model are provided in Supplementary Table 2. ICCs were also calculated for this adjusted model to evaluate how much between-subject variance remained after accounting for physiological covariates.

## Results

Of 47 screened volunteers, 25 participants were enrolled (13 females, 12 males); exclusions were primarily for pre-existing somatic or psychiatric conditions. All 25 completed the protocol (Fig. 1). Descriptive statistics during baseline for both conditions are presented in Table 1.

**Table 1:** Descriptive statistics. Data are presented as mean ± SD for symmetric variables, median [IQR] for skewed variables, or count (%) for categorical variables, measured at resting baseline prior to intervention

Demographics	Distribution		
Sex (Female/Male)	13 (52%) / 12 (48%)		
Age (years)	23 [21–25]		
Body Mass Index (BMI, kg/m^2^)	23.7 ± 1.8		
**Primary Outcome**	**Placebo** **Baseline Distribution**	**Endotoxemia** **Baseline** **Distribution**	***p*-value from Baseline comparison **^**a**^
Finger qCRT (s)	2.4 [1.3–5.8]	1.6 [0.9–6.1]	0.745
**Covariates**	**Placebo** **Baseline Distribution**	**Endotoxemia** **Baseline** **Distribution**	***p*-value from Baseline comparison **^**a**^
Heart Rate (BPM)	62.7 ± 12.0	62.6 ± 8.1	0.947
Systolic BP (mmHg)	114.7 ± 9.0	116.7 ± 8.9	0.454
Diastolic BP (mmHg)	74.0 [72.0—77.0]	75.0 [71.0—79.0]	0.170
IL-6 (pg/mL)	3.5 [3.5—3.5]	3.5 [3.5—3.5]	1.000
Tympanic Temperature (°C)	36.6 ± 0.3	36.6 ± 0.4	0.844
Finger Skin Temperature (°C)	29.8 [26.7—32.3]	29.4 [24.2—32.4]	0.475

**Fig. 2:**
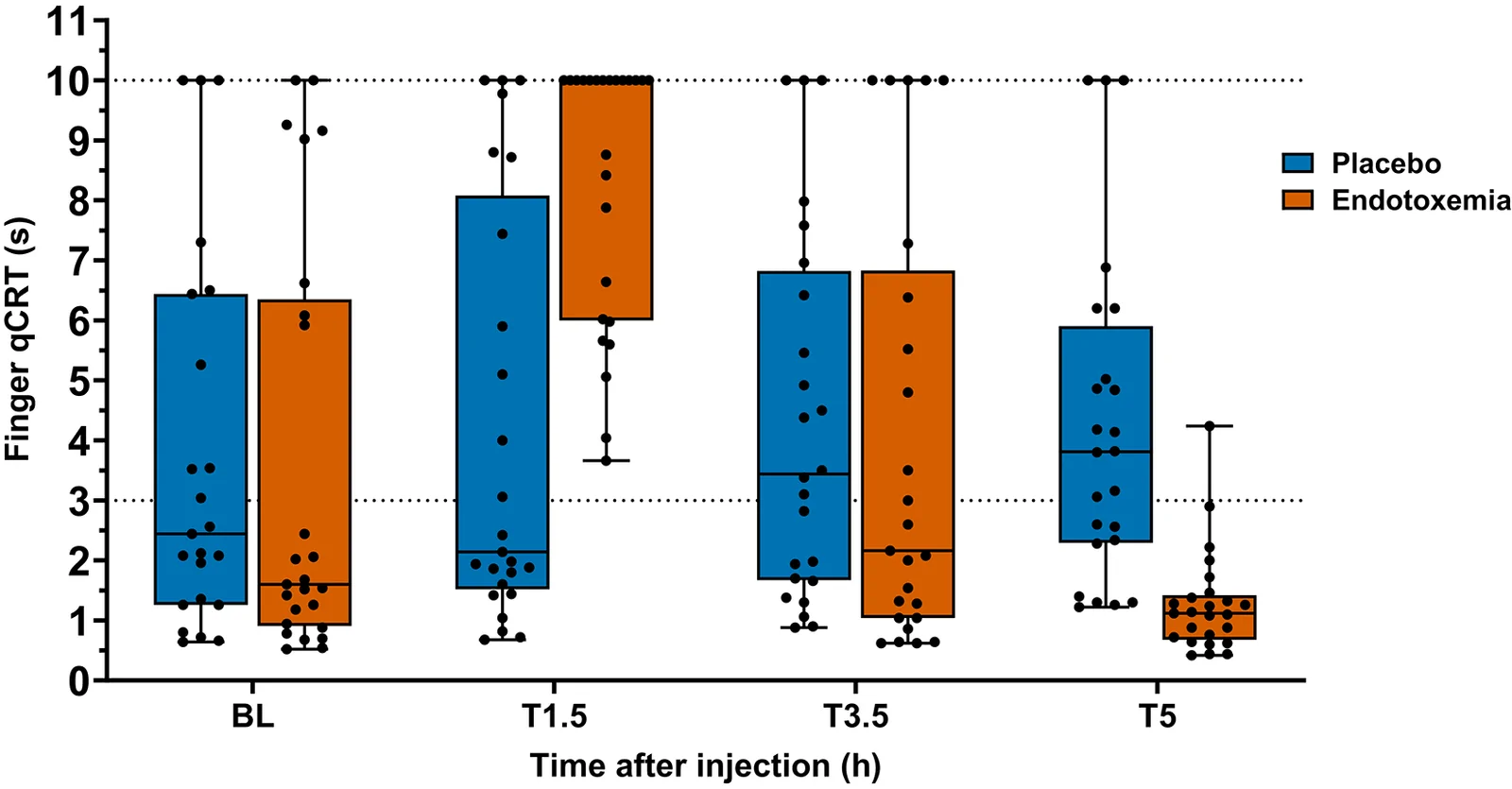
Descriptive boxplots show the distribution of raw data quantitative capillary refill time (qCRT) measured on the finger pulp for placebo (blue) and experimental endotoxemia (orange) at baseline (BL) and each time point (T hours) after injection. qCRT values exceeding the measured maximum of 10 s were assigned 10 s (number of qCRT values > 10 s per time point and condition: placebo BL: 3, T1.5: 3, T3.5: 3, T5: 3; endotoxemia BL: 2, T1.5: 14, T3.5: 5, T5: 0). Individual data points are overlaid. Boxes represent interquartile range (IQR), black horizontal lines indicate medians, and whiskers indicate minimum and maximum values

Normality was evaluated at baseline using the Shapiro–Wilk test. Graphical presentation of full longitudinal descriptive statistics for quantitative capillary refill time (qCRT) is provided in Fig. 2, and for the covariates in Supplementary Fig. 1. ^a^Baseline comparison between placebo and endotoxemia was calculated using paired t-tests for normally distributed parameters and Wilcoxon signed-rank tests for skewed parameters

In the primary unadjusted model (m0), pairwise contrasts of estimated marginal means revealed a distinct biphasic trajectory under endotoxemia. Compared to placebo, finger qCRT was significantly prolonged at 1.5 h post-injection (absolute difference: + 4.13 s, p < 0.0001) and significantly shortened at 5 h post-injection (absolute difference: –3.10 s, p < 0.0001) (Table 2, Fig. 2). The qCRT values in the placebo condition were stable across all time points (Table 2, Fig. 2) but showed substantial variance. Finger measurements were right-censored at 10 s: at 1.5 h under endotoxemia, 14 of 25 participants (56%) reached the ceiling. The underlying fixed-effect parameter estimates from the primary model are provided in Supplementary Table 1. The intraclass correlation coefficient (ICC) was 30%.

**Table 2:** Finger quantitative capillary refill time (qCRT) and pairwise condition contrasts over time, derived from the primary Tobit mixed-effects model (m0)

**Time point**	**Mean (Model-Predicted)**			
	**Endotoxemia**	**Placebo**	**Difference (Endotoxemia − Placebo)**	**p-value**
Baseline (BL)	3.51 s	3.61 s	-0.10 s (95% CI: -1.47, 1.27)	0.8897
1.5 Hours (T1.5)	8.31 s	4.18 s	+ 4.13 s (95% CI: 2.79, 5.47)	< 0.0001
3.5 Hours (T3.5)	3.96 s	4.32 s	-0.36 s (95% CI: -1.72, 0.99)	0.5985
5.0 Hours (T5)	1.27 s	4.38 s	-3.10 s (95% CI: -4.46, -1.75)	< 0.0001

Estimates and differences are derived as Estimated Marginal Means (EMMs) from the primary Tobit mixed-effects model m0 to account for right-censoring at 10.0 s

**Fig. 3:**
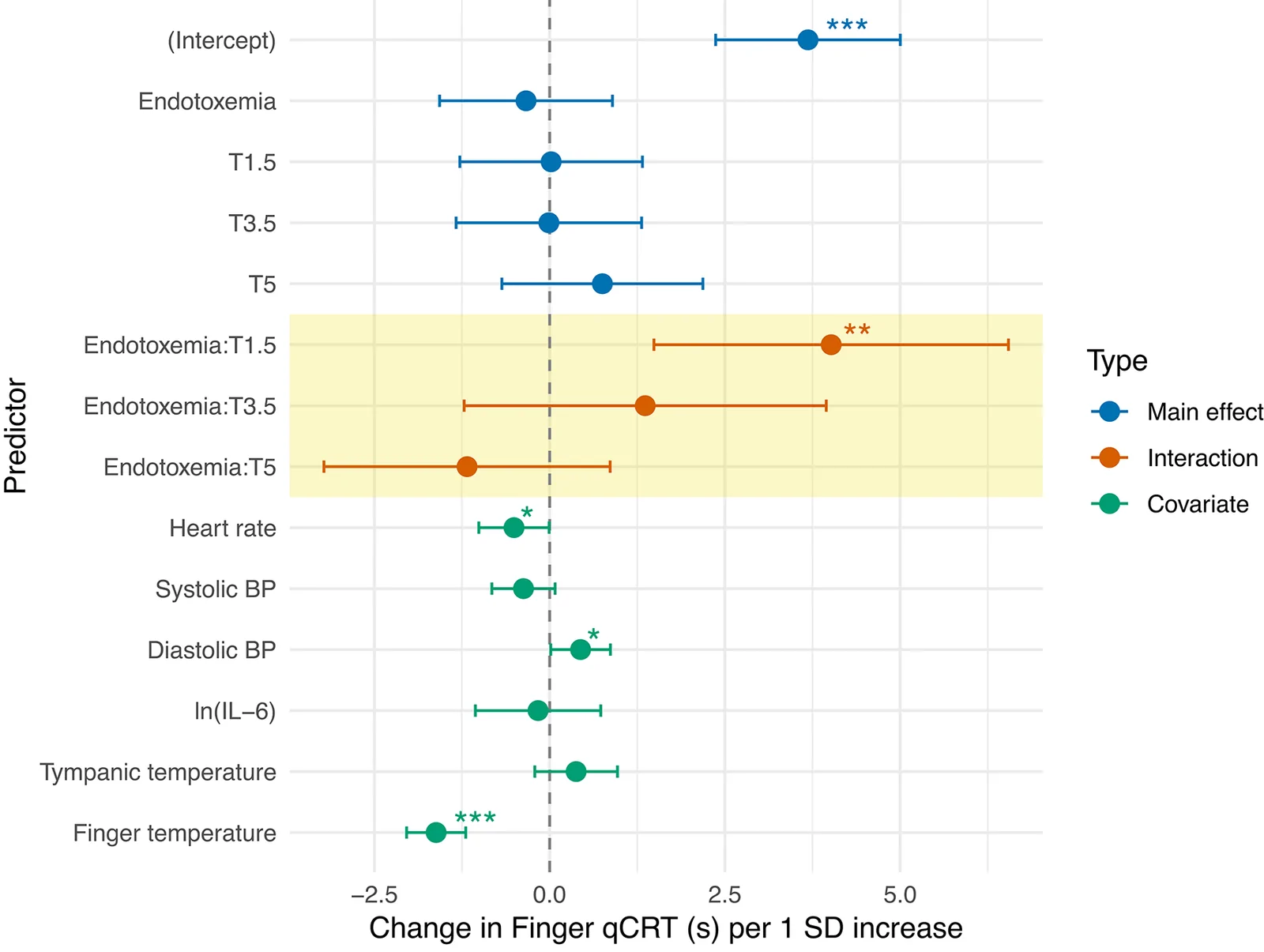
Coefficient plot of fixed effects derived from the secondary, exploratory Tobit mixed-effects model (m1) for finger quantitative capillary refill time (qCRT). Predictors are shown on the y-axis and grouped by type: main effects (blue); interaction terms (orange); covariates (green). Continuous covariates were standardized (z-scored), such that coefficients represent the estimated change in qCRT (β, in seconds) per SD increase on the x-axis. Global Means and Standard Deviations used as scaling factors are provided in Supplementary Table 3. Horizontal lines indicate 95% confidence intervals for each coefficient. Significant values are indicated as *p < 0.05, **p < 0.01, ***p < 0.001, and ·p ≤ 0.1 indicates trend-level association

In the exploratory, covariate-adjusted model (m1), multicollinearity was minimal; excluding structural interaction effects, all physiological covariates returned VIFs < 5. The prolongation finger qCRT at 1.5 h post-injection remained significant after adjusting for heart rate, systolic BP, diastolic BP, tympanic temperature, local skin temperature, and IL-6 plasma concentration (Fig. 3; full model parameters in Supplementary Table 2). However, the observed reduction in qCRT values 5 h post-injection was attenuated when adjusting for covariates, and no longer significant (Fig. 3; full model parameters in Supplementary Table 2). Local skin temperature was the dominant covariate: each SD increase was associated with a 1.62 s reduction in qCRT. Heart rate was negatively associated with finger qCRT and diastolic BP was positively associated; both narrowly met the prespecified α = 0.05 threshold. The ICC fell to 16% in m1.

## Discussion

We found that finger qCRT followed a clear biphasic course during experimental endotoxemia. While resting measurements showed high inter-individual variability, the prolongation at 1.5 h shifted all participants well beyond the 3-s threshold which is often suggested as a resuscitation target. Conversely, during fever resolution (5 h), qCRT fell below both baseline and the clinical threshold to a mean of approximately 1.3 s, shifting most participants into a hyperdynamic range despite persistent systemic inflammation. We did not measure skin blood flow or sympathetic activity, and any mechanistic interpretation therefore remains provisional. The biphasic pattern is nonetheless consistent with two established responses: vasoconstriction during fever induction, which would be expected to reduce peripheral perfusion, and thermoregulatory vasodilation during fever resolution (Supplementary Fig. 1), which would be expected to shorten cutaneous capillary refill time [29, 30, 34]. The late shortening could equally represent transient thermoregulatory hyperaemia unrelated to recovery. The early prolongation, occurring despite preserved systemic blood pressure, may reflect alterations in critical closing pressure or vascular waterfall mechanics, as previously speculated [14]. At 1.5 h, 14 of 25 participants reached the 10-s measurement ceiling; Tobit modelling accounts for this censoring to provide an unbiased estimate of the true latent prolongation.

Resting finger qCRT showed an intraclass correlation coefficient of 30% in the unadjusted model (m0), meaning that between-person differences account for only a minority of total variance. In this experimental setting, a single measurement taken without a prior baseline could not therefore be reliably interpreted against population-level reference ranges as within-subject variability dominates the measurement. In contrast, the intraindividual trajectory is more informative. The same participants who varied widely at rest showed a consistent, directional response to endotoxemia, often with more than 4 s of change across a single inflammatory episode. Thus, our findings indicate that qCRT tracks the inflammatory response trajectory, lengthening as inflammation peaks and shortening as it resolves (Fig. 2, Table 2, Supplementary Fig. 1). The consequence of this is that qCRT in this model is, as a metric, more appropriately understood as a monitoring tool for the inflammatory trajectory rather than as a static triage classifier applied at a single time point, or in relation to a static reference value. Quantitative measurement further addresses the observer variability that is known to limit visual CRT reproducibility [15, 17].

Our data provide some insight into the underlying physiology of capillary refill. In the covariate-adjusted model (m1), the net prolongation at 1.5 h remained significant after adjustment for tympanic temperature, skin temperature, blood pressure, heart rate, and IL-6. This could indicate that early prolongation might reflect a primarily local vascular mechanism, such as altered arteriolar tone or critical closing pressure [14], rather than the concurrent systemic changes we adjust for in this model. At 5 h, the between-condition difference was no longer significant, indicating that late shortening is strongly mediated by the thermoregulatory and hemodynamic covariates included in the model. Skin temperature was the dominant physiological mediator: each standard-deviation increase was associated with a 1.62 s reduction in finger qCRT, consistent with the known temperature dependence of capillary refill [19, 20]. We recognize that skin temperature serves as one of the primary drivers of cutaneous vascular tone. By including it as a covariate in this model we did not intend to portray it as a confounder of qCRT but rather explore its magnitude as a mediator in this model. Regardless, in febrile individuals, this implies that skin temperature modifies cutaneous microvascular responsiveness through physiological thermoregulation. Thus, standardized measurement conditions and careful consideration of local skin temperature at the time of measurement are important for both the interpretation of CRT and the understanding of its underlying physiological mechanisms.

Over the past decade, capillary refill time has reemerged as a clinical tool of interest. The ANDROMEDA-SHOCK trials represent the most extensive clinical trials for CRT-guided resuscitation. The first ANDROMEDA-SHOCK Randomized Clinical Trial found no significant 28-day mortality difference between CRT-guided and lactate-guided strategies (34.9% vs. 43.4%, p = 0.06) [9], though it demonstrated significant, positive impact on prespecified secondary outcomes including reduced organ dysfunction, supported by subsequent Bayesian analyses [8]. Recently, the follow-up trial, the ANDROMEDA-SHOCK-2 Randomized Clinical Trial, reported superiority on a hierarchical composite outcome (win ratio 1.16, p = 0.04), driven largely by reduced organ support duration [21]. In clinical practice, CRT is rarely interpreted in isolation. It is integrated with perfusion pressure, cardiac output, vasopressor use, lactate, body and skin temperature, and its evolution over time.

Our study contributes to the physiological understanding of qCRT rather than to this clinical decision framework. We did not measure skin blood flow directly, whether by laser Doppler flowmetry or perfusion imaging, and we therefore address a narrower question: if we consider qCRT as a marker of peripheral hypoperfusion or microcirculatory dysfunction, how much of the qCRT signal in febrile individuals reflects systemic microvascular perfusion, and how much reflects local thermoregulatory dynamics?

Under controlled inflammation in the absence of shock, qCRT varied considerably between individuals at rest, was strongly associated with local skin temperature in the adjusted model and moved biphasically across a range that spanned the commonly used clinical threshold twice. This does not invalidate the clinical utility or prognostic value demonstrated in the ANDROMEDA trials. It does show, however, that even under a low-to-moderate endotoxin dose in a controlled setting, qCRT is a dynamic signal influenced by factors beyond, and partly decoupled from, systemic haemodynamics alone. Together with other recent physiological work [11, 13, 14], these findings indicate that the mechanisms underlying qCRT are not reducible to a single haemodynamic determinant.

This study has several key limitations to acknowledge. The 25 participants were healthy young adults; findings most likely represent a healthy response to experimental human endotoxemia and transient, systemic inflammation, which is a homogeneous, TLR-4-driven response that does not replicate the pathophysiological complexity of clinical sepsis or septic shock [35, 36]. Therefore, the findings cannot be directly extrapolated to patients with septic shock who exhibit vasoplegia, vasopressor dependency, altered or depressed cardiac output, tissue hypoxia, severe endothelial dysfunction, and impaired microvascular autoregulation. Additionally, mechanistic explanations regarding vascular tone and blood flow distribution remain speculative and hypothesis generating, as direct measurements of skin blood flow (e.g., laser Doppler flowmetry or perfusion imaging), sympathetic nerve activity, or vascular resistance were not obtained. Finally, qCRT measured via optical spectroscopy is not directly equivalent to observer-based visual CRT.

In conclusion, finger qCRT tracks the inflammatory trajectory in a standardized human model of acute systemic inflammation: prolonged during peak inflammation, shortened during resolution, and with intraindividual changes often exceeding 4 s. The high resting-state variability we observed highlights that within-subject changes across time are particularly informative when evaluating qCRT dynamics and shows that qCRT cannot be interpreted as a diagnostic marker against a fixed threshold in an individual patient, since between-subject differences account for only a minority of total variance. It is instead more useful for tracking change within a patient, where the biphasic response to inflammation is large and reproducible. Skin temperature acts as one of the primary determinants of CRT in our model, demonstrating that physiological thermoregulation alone can significantly shift refill kinetics. While these physiological observations in healthy participants cannot redefine clinical practice in septic shock, they emphasize the importance of accounting for skin temperature and the dynamic individual baseline. Prospective studies in sepsis populations, combining quantitative CRT measurements, standardized temperature tracking, and comprehensive hemodynamic monitoring, are needed to further clarify these microcirculatory mechanics in a clinical setting.

## Supplementary Information

Supplementary Material 1.

## Data Availability

The de-identified data that support the findings of this study are available from the corresponding author upon reasonable request.
