# Supplementary material for: Physiological limits of capillary refill time: a human endotoxemia study
Source: Crit Care. 2026 Aug 31;30:461. doi: 10.1186/s13054-026-06247-8 (PMC13548548; doi:10.1186/s13054-026-06247-8)
Supplement: Supplementary file 1 [file 13054_2026_6247_MOESM1_ESM.docx]

**Supplementary Material 1**

Supplementary Figure 1: Supporting line plots with Mean and standard deviation (SD, showing placebo in blue and endotoxemia in red, for all included covariates; **A**: Systolic blood pressure, **B**: Diastolic blood pressure, **C**: Heart rate, **D**: ln-transformed interleukin 6 (Il-6) blood concentration (Median [IQR] to account for non-normal distribution and for ease of interpretation. Ln-transformed IL-6 values were used in linear-mixed effects model (m1).), **E**: Finger skin temperature, **F**: Sternum skin temperature, **G**: Tympanic temperature. Dotted vertical lines indicates capillary refill measurement occasions.

**Supplementary Material 2**

List of Inclusion and Exclusion Criteria

Inclusion criteria:
- Healthy
- 18-40 years-old
- Consent form signed
- Having a Swedish personal identification number (personnummer)

Exclusion criteria (all medical criteria assessed by an MD):
- Non-fluent in Swedish
- Unable to read
- Unable or unwilling to provide informed consent
- BMI below 18.5 or above 30 kg/m^2^
- Smoking
- Excessive alcohol consumption
- Pregnancy or breastfeeding
- Positive on drug screening
- Current somatic disease including skin disease affecting the measurement areas
- Current depression, anxiety, sleep disturbances or other psychiatric illnesses
- Abnormal blood analyses: abnormal clinical levels in blood cell counts, haemoglobin, sodium, potassium, chloride, calcium, phosphate, creatinine, blood urea nitrogen, uric acid, glucose, aspartate aminotransferase, alanine aminotransferase, gamma-glutamyl transpeptidase, bilirubin, thromboplastin time and partial thromboplastin time
- High-sensitivity C-reactive protein > 10 mg/L.
- History of cardiovascular, pneumological, immune disease
- Moderate viral or bacterial infection in the past two weeks
- Severe and/or long-term COVID-19: positive SARS-CoV-2 PCR-test or antigen test and/or history of symptoms since February 2020 indicative of infection with SARS-CoV-2 (fever, cough, sore throat, runny or blocked nose, nausea, muscle pain, shortness of breath), which was of high intensity and/or of long duration (≥ 8 weeks).
- Recent vaccination (within previous two weeks)
- Abnormal cardiovascular measurements
- Current or previous cancer
- Any concomitant medication other than contraceptives (14 days prior to the start and throughout the study period)
- Shift work or irregular sleep-wake cycle (within 14 days of the study period)
- Needle anxiety or blood phobia
- Invisible veins in the antecubital area of the arms or very difficult blood sampling (self-reported or experienced during screening).
- Previous participation in a study involving administration of LPS

**Supplementary Material 3**

Supplementary Table 1: Full fixed-effect parameter estimates for the primary finger qCRT Tobit mixed-effects model (m0). The coefficients (β, given in absolute seconds) represent the raw fixed-effect parameters of the model rather than the post-estimation marginal means presented in the main text. The model included fixed effects of Condition (placebo vs. endotoxemia), Timepoint (BL, T1.5, T3.5, T5), and their interaction (Condition × Timepoint), with random intercepts for each subject. This way, the intercept represents the estimated qCRT at baseline under placebo. Main effects of Timepoint indicate the change from baseline within the placebo condition, while interaction terms represent the additional change under endotoxemia relative to the placebo baseline. Estimates are shown with 95% confidence intervals (CI) and p-values. Significant values are indicated as *p < 0.05, **p < 0.01, ***p < 0.001.

| **Predictor** | **β (s)** | **95% CI** | **p** |
| --- | --- | --- | --- |
| Intercept | 3.61 | [2.40, 4.81] | <0.001 *** |
| Endotoxemia | -0.10 | [-1.51, 1.31] | 0.892 |
| T1.5 | 0.57 | [-0.84, 1.98] | 0.425 |
| T3.5 | -0.71 | [-0.72, 2.14] | 0.326 |
| T5 | 0.77 | [-0.66, 2.19] | 0.289 |
| Endotoxemia: T1.5 | 4.22 | [2.25, 6.20] | <0.001 *** |
| Endotoxemia: T3.5 | -0.27 | [-2.25, 1.72] | 0.791 |
| Endotoxemia: T5 | -3.01 | [-4.99, -1.02] | 0.003** |

Supplementary Material 4

| **Predictor** | **β (s)** | **95% CI** | **p** |
| --- | --- | --- | --- |
| Intercept | 3.68 | [2.37, 5.00] | <0.001 *** |
| Endotoxemia | -0.34 | [-1.57, 0.90] | 0.593 |
| T1.5 | 0.02 | [-1.28, 1.32] | 0.974 |
| T3.5 | -0.01 | [-1.33, 1.31] | 0.987 |
| T5 | 0.75 | [-0.68, 2.19] | 0.304 |
| Endotoxemia: T1.5 | 4.02 | [1.49, 6.54] | 0.002 ** |
| Endotoxemia: T3.5 | 1.36 | [-1.22, 3.95] | 0.301 |
| Endotoxemia: T5 | -1.18 | [-3.22, 0.86] | 0.258 |
| Heart rate | -0.51 | [-1.01, -0.01] | 0.047 * |
| Systolic BP | -0.37 | [-0.82, 0.08] | 0.105 |
| Diastolic BP | 0.44 | [0.02, 0.87] | 0.042 * |
| ln(IL-6) | -0.17 | [-1.06, 0.73] | 0.718 |
| Tympanic temperature | 0.38 | [-0.21, 0.97] | 0.209 |
| Skin site temperature | -1.62 | [-2.04, -1.12] | <0.001 *** |

Table 2: Tobit mixed-effects model (m1) estimates for finger quantitative capillary refill time (qCRT). Fixed-effect estimates (β, in seconds) for qCRT measured at the finger and sternum during placebo and experimental endotoxemia. The model included fixed effects of Condition (placebo vs. endotoxemia), Timepoint (BL, T1.5, T3.5, T5), their interaction (Condition × Timepoint), with random intercepts for each subject. Continuous covariates were standardized (z-scored), such that coefficients represent the estimated change in qCRT (β, in seconds) per standard deviation increase. Estimates are shown with 95% confidence intervals (CI) and p-values. Significant values are indicated as *p < 0.05, **p < 0.01, ***p < 0.001, indicates trend-level association.

Supplementary Material 5

Supplementary Table 3: Global Scaling Parameters for converting m1 model’s (see Supplementary Table 2) standardized regression coefficients ($\beta$) back into original physical units and mean ± SD for each condition and time point.

|  |  | **(Mean ± SD)** | | | | |
| --- | --- | --- | --- | --- | --- | --- |
| **Covariates** | **Global Scaling Parameters (Mean ± SD)** | **Condition** | **Baseline (BL)** | **T 1.5** | **T 3.5** | **T 5** |
| Heart Rate (BPM) | 70.0 ± 13.0 | Placebo | 62.7 ± 12.0 | 66.9 ± 10.1 | 62.0 ± 9.6 | 68.0 ± 10.7 |
|  |  | Endotoxemia | 62.6 ± 8.1 | 73.1 ± 11.5 | 81.8 ± 10.2 | 83.0 ± 11.5 |
| Systolic BP (mmHg) | 114.0 ± 9.3 | Placebo | 114.7 ± 9.0 | 110.1 ± 8.9 | 110.6 ± 8.2 | 115.8 ± 7.7 |
|  |  | Endotoxemia | 116.7 ± 8.9 | 114.6 ± 11.9 | 112.5 ± 9.8 | 114.8 ± 8.6 |
| Diastolic BP (mmHg) | 72.2 ± 8.1 | Placebo | 73.9 ± 6.5 | 69.6 ± 5.8 | 73.0 ± 6.4 | 74.1 ± 6.6 |
|  |  | Endotoxemia | 75.8 ± 6.8 | 70.8 ± 13.0 | 68.3 ± 7.8 | 71.3 ± 8.3 |
| Tympanic  Temperature (°C) | 37 ± 0.5 | Placebo | 36.6 ± 0.3 | 36.8 ± 0.4 | 36.9 ± 0.3 | 36.9 ± 0.4 |
|  |  | Endotoxemia | 36.6 ± 0.4 | 37.0 ± 0.5 | 37.8 ± 0.3 | 37.5 ± 0.4 |
| Finger Skin Temperature (°C) | 28.0 ± 3.9 | Placebo | 28.9 ± 3 | 27.0 ± 3.6 | 27.6 ± 3.7 | 28.0 ± 3.3 |
|  |  | Endotoxemia | 28.2 ± 4.2 | 24.4 ± 2.1 | 29.3 ± 4 | 31.0 ± 2.7 |
| IL-6 (pg/mL) (Median [IQR]) ^a^ | 3.1 ± 1.7^b^ | Placebo | 3.5 [3.5-3.5] | 6.3 [3.9-10.3] | 7.0 [5.2-10.8] | 11.0 [5.7-24.5] |
|  |  | Endotoxemia | 3.5 [3.5-3.5] | 218.0 [179.7-366.5] | 215.5 [149-289] | 38.0 [19-79] |
